# Granulomatous *Pneumocystis jiroveci* Pneumonia in an HIV-Positive Patient on Antiretroviral Therapy: A Diagnostic Challenge

**DOI:** 10.2174/1874306402115010019

**Published:** 2021-06-18

**Authors:** Montserrat Diaz-Abad, Kathryn S. Robinett, Anayansi Lasso-Pirot, Teklu B. Legesse, Mariam Khambaty

**Affiliations:** 1 Department of Medicine, University of Maryland School of Medicine, Baltimore, Maryland, USA; 2 Department of Pediatrics, University of Maryland School of Medicine, Baltimore, Maryland, USA; 3 Department of Pathology, University of Maryland School of Medicine, Baltimore, Maryland, USA

**Keywords:** *Pneumocystis jiroveci* pneumonia, Human immunodeficiency virus, Granulomatous, Diagnosis, Biopsy, Antiretroviral therapy, Lung mass

## Abstract

Human Immunodeficiency Virus (HIV)-related Opportunistic Infections (OI), including *Pneumocystis jiroveci* pneumonia (PCP), have become much less commonplace with anti-retroviral therapy (ART). Despite this, OIs are still common and it is important to remain vigilant for their presence and be aware of how ART and OI chemoprophylaxis may lead to atypical disease presentations. We present the case of a 51-year-old woman with HIV and CD4+ T helper lymphocytes cell count > 200 cells/ul on both ART and trimethoprim/sulfamethoxazole prophylaxis who presented with cavitating lung masses, mediastinal lymphadenopathy and pleural effusions. Negative bronchoalveolar lavage (BAL) and transbronchial biopsy (TBBx) prompted a second diagnostic procedure with a transthoracic core needle biopsy; the final diagnosis was granulomatous PCP. This case showcases a very rare presentation of PCP, with both large cavitating lung masses on imaging and granulomatous reaction on pathology, as well as the challenge of a potentially missed diagnosis with negative BAL and TBBx requiring transthoracic core needle biopsy for a final diagnosis.

## 1 INTRODUCTION

Being the most common cause of death in human immunodeficiency virus (HIV)-related diseases, opportunistic infections (OI), including *Pneumocystis jiroveci* pneumonia (PCP), are becoming much less commonplace with the advent of potent and more readily available anti-retroviral therapy (ART). Prior to the availability of highly-active ART and PCP prophylaxis, it is estimated that up to 75% of people living with AIDS developed PCP in the US [1]. That number fell precipitously throughout the decades from 3.5 between 2003 to 2007 [2] to less than 1 case per 100 person-years [3]. In 2017, an estimated 10,590 patients were hospitalized with PCP [4]. Indeed, the spectrum of HIV-related diseases that patients are hospitalized for is very different compared to 10 or 15 years ago, with prolonged patient survival now reported due to improved medication efficacy and decreased toxicities [5].

Despite this, OIs are still common and it is important to remain vigilant for their presence and be aware of how ART and OI chemoprophylaxis may contribute to atypical disease presentations. We present the case of a 51-year-old woman with HIV and CD4+ T helper lymphocytes (CD4) cell count > 200 cells/ul, on both ART and trimethoprim/sulfamethoxazole (TMP/SMX) chemoprophylaxis, who presented with cavitating lung masses, mediastinal lymphadenopathy and pleural effusions, and was diagnosed with granulomatous PCP with transthoracic core needle biopsies, a very rare presentation of this infection.

## 2 CASE PRESENTATION

A 51-year-old woman was admitted with 3 weeks of cough, initially dry then productive of a small amount of clear sputum, and 6 days of subjective fevers, chills and dyspnea on exertion. On the day prior to admission, she experienced worsening malaise, fatigue and dyspnea. Her past medical history included HIV infection diagnosed 2 years earlier, with a CD4 cell count of 220 cells/ul measured 2 months prior to admission, cervical dysplasia with cone biopsy 8 months earlier and chronic anemia. She reported no recent travel history or sick exposures. Her ART regimen was lamivudine/zidovudine plus lopinavir/ritonavir, with TMP/SMX 800/160 mg 3 times weekly for PCP prophylaxis.

On physical examination, the patient was in no acute distress. She was febrile at 102 °F, pulse was 84 beats/minute, and respirations were 22 breaths/minute. Blood pressure was 86/50 mm Hg and oxygen saturation was 99% on room air. Lungs were clear, with bilateral breath sounds. There was no palpable lymphadenopathy. Laboratory values were significant for a lactate dehydrogenase level of 217 U/L (100-190), albumin 2.0 g/dl, hemoglobin 10.7g/dl, and white blood cell count 7.9 cells/ul, with differential: 71.1% neutrophils, 15.9% lymphocytes, 12.1% monocytes, 0.4% eosinophils and 0.8% basophils.

**Fig. (1):**
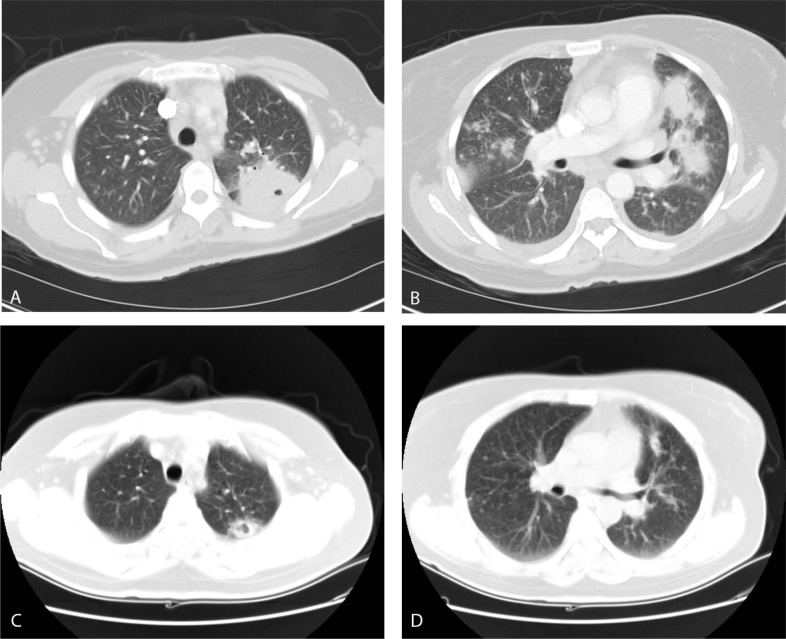
Admission Computed Tomography scan (CT) of the chest, showing: (**A**) Pleural based large cavitating lung mass in the left upper lobe measuring 4.2 x 4.2 cm; and (**B**) Multiple masses and nodules scattered throughout both lungs. CT of the chest 3 months later, showing: (**C**) Persistent but much smaller cavitating left upper lobe nodule, and (**D**) Nearly complete resolution of the previous nodules and masses, with multiple peripheral bilateral sub-cm non-calcified pulmonary nodules.

Computed Tomography (CT) of the chest revealed subcarinal, prevascular and right hilar lymphadenopathy. Multiple masses and nodules were scattered throughout both lungs, the largest cavitating and abutting the major fissure in the left upper lobe measuring 4.2 x 4.2 cm, with another large mass at the right cardiophrenic angle measuring 2.2 x 4.6 cm. There were also bilateral pleural effusions with associated compressive atelectasis of the lower lobes and fibrotic changes in the left apical region, (Fig. **1**).

The patient was admitted with the diagnosis of pneumonia of uncertain etiology, placed on airborne isolation for tuberculosis, and was started on ceftriaxone 1g Intravenous (IV) daily, azithromycin 500 mg IV daily and TMP/SMX 2 double strength tablets orally every 8 hours. Sputum Gram stain and bacterial culture, 3 Acid-Fast Bacilli sputum smears and purified protein derivative testing were negative. The patient continued to have intermittent fevers 101-102 °F for the next 4 days, then became afebrile and started feeling overall better. She experienced nausea and vomiting as a side effect of TMP/SMX.

**Fig. (2):**
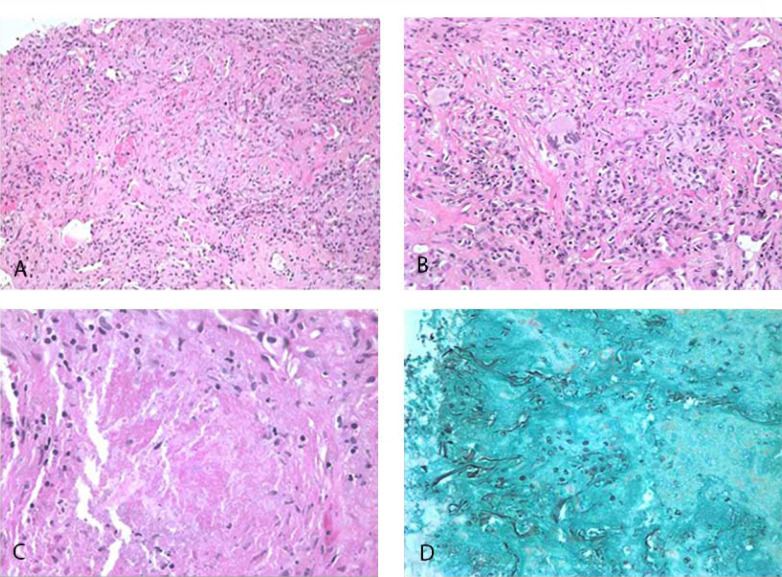
Transthoracic core needle biopsies histology. (**A**) Granulomatous inflammation with epithelioid cell aggregates and lymphoplasmacytic infiltrates. The granulomas in some areas are not well-formed (Hematoxylin and Eosin, Low magnification). (**B**) Granulomatous inflammation with aggregates of epithelioid cells and scattered Langhans type multinucleated giant cells (Hematoxylin and Eosin, medium magnification). (**C**) Necrotic center of the granuloma with surrounding loose epithelioid cell aggregates (Hematoxylin and Eosin, High magnification). (**D**) Scattered cysts of *Pneumocystis jiroveci* in the center of necrotic granuloma (Gomori methenamine silver, high magnification).

Five days after admission, the patient underwent fiberoptic bronchoscopy under moderate sedation. There were no abnormalities in the airways, and Bronchoalveolar Lavage (BAL) of the lingula as well as 5 Transbronchial Biopsies (TBBx) of the left upper lobe were performed. All cultures and smears were negative. Cytology was negative for malignancy and PCP. The TBBx of the left upper lobe showed benign bronchial tissue and a fragment of lung parenchyma with interstitial fibrosis. TMP/SMX was discontinued in view of negative results for PCP while azithromycin and ceftriaxone were continued. Three days later, fever and malaise restarted, and because of suspicion for nocardiosis, TMP/SMX was restarted. CT of the head showed maxillary, frontal and ethmoid sinus disease, without other pathology. That day, 2 transthoracic core needle biopsies were performed in the left upper lung cavitating pleural-based mass. Pathology revealed lung parenchyma and bronchial tissue with acute inflammation and fibrosis, and granulomas with coagulation necrosis with organisms within morphologically consistent with *Pneumocystis jiroveci* noted on Gomori methenamine silver (GMS) stain by direct microscopy, and negative Acid-Fast Bacilli stain, (Fig. **2**).

Azithromycin and ceftriaxone were stopped. The patient was restarted on oral TMP/SMX and again became afebrile; malaise and fatigue improved. Due to persistent nausea and vomiting, therapy was switched to IV formulation. She continued to improve and felt back to baseline and was discharged 1 week later on oral clindamycin and primaquine. CT of the chest 3 months later revealed nearly complete resolution of the previous findings, with a significantly improved cavitating left upper lobe nodule, multiple peripheral bilateral sub-cm non calcified pulmonary nodules, and resolution of bilateral pleural effusions and mediastinal lymphadenopathy (Fig. **1**).

## 3 RESULTS AND DISCUSSION

The present case showcases a very rare presentation of PCP, with large cavitating lung masses, mediastinal lymphadenopathy and pleural effusions on CT imaging and granulomatous reaction on pathology, as well as the challenge of a potentially missed diagnosis, with both negative BAL and TBBx requiring transthoracic core needle biopsy for final diagnosis. The patient had a moderate level of immunity and was also on both ART and PCP chemoprophylaxis, which likely contributed to this atypical presentation.

The BAL and TBBx failed to reveal the *Pneumocystis jiroveci* organisms in this case. While BAL and TBBx have been reported to be highly sensitive for this organism, and the diagnostic yield for TBBx has been reported as 97% [6], patients who have failed PCP chemoprophylaxis have a smaller burden of organisms and an increased frequency of false-negative results on BAL [7]. The patient required transthoracic core needle biopsy for the diagnosis, and at one moment, the appropriate therapy with TMP-SMX was stopped due to the unlikely possibility of PCP with the prior negative results, with prompt recurrence of symptoms. There was almost complete resolution of the CT findings with therapy, which points to appropriate diagnosis and therapy.

This patient presented with lung masses, nodules and cavitation, mediastinal lymphadenopathy and pleural effusions, a presentation that is very uncommon. The classic radiographic appearance of PCP pneumonia is that of bilateral alveolar infiltrates that begin in the perihilar areas and progress to diffusely involve the lungs, and CT of the chest usually shows ground-glass attenuation or opacities. Atypical presentations of PCP on CT are less common and include focal, segmental or lobar consolidation, nodules and cavitating lesions, a military pattern, pleural effusions, lymphadenopathy, cystic spaces, bullae and pneumothorax [8-13].

The biopsy, in this case, reported granulomatous lesions with caseating necrosis with the organisms within. The typical histologic appearance of PCP consists of intra-alveolar foamy eosinophilic exudates, which contain trophozoites and cysts of the organism. The atypical granulomatous reaction can be proven only by finding the cysts on Gomori’s Methenamine Silver staining in the absence of other organisms on appropriate stains and cultures [14, 15]. Granulomatous inflammation in PCP is unusual [11, 12]. It can be seen in about 5% of patients, usually when immunodeficiency is more limited [15], and can be seen on CT of the chest as a solitary nodule, multiple nodules of varying sizes, or even a mass mimicking lung carcinoma [16]. PCP with a granulomatous reaction has also been reported as part of an immune reconstitution inflammatory syndrome [15, 17, 18]. In granulomatous PCP without alveolitis, BAL may be negative because the cysts are trapped in the necrosis of the granulomas, or there are fewer organisms in the granulomas. Patients with HIV have a T-cell deficiency and, due to this, have difficulty in mounting a granulomatous reaction [19]. It is possible that the relatively lower immunodeficiency in this case -CD4 cell count of 220 cell/ul- allowed for the development of the granulomatous reaction.

There have been several reports of unusual presentations of PCP in patients with HIV. One case had pleural based masses, nodules and mediastinal lymphadenopathy in a patient on chemoprophylaxis but without ART or a granulomatous reaction. The diagnosis was made initially by transthoracic fine needle biopsy [20]. A patient on ART but not compliant with chemoprophylaxis presented with multiple pulmonary nodules. BAL and TBBx were negative, and the diagnosis was made with a thoracoscopic lung biopsy showing well-formed necrotizing granulomas with *Pneumocystis* organisms [21]. In another case, the patient presented after starting ART with multiple lung nodules, some cavitating, CD4 count > 250 cell/ul and off chemoprophylaxis. BAL, TBBx and transthoracic needle biopsy were negative, requiring a thoracoscopic lung biopsy that revealed necrotizing granulomas containing *Pneumocystis* organisms [17].

To our knowledge, this is the first case of PCP that reunites together in one patient all these atypical CT imaging and histologic findings, along with both a negative BAL and TBBx while receiving ART and chemoprophylaxis at the time of admission and with a CD4 count > 200 cell/ul. PCP should be in the differential diagnosis of patients with HIV who present with a lung mass. There have been increasing numbers of unusual presentations of PCP in HIV patients, leading to delayed diagnosis and appropriate treatment [22]. The spectrum of imaging abnormalities associated with PCP now includes abnormalities of the lung parenchyma, airways, lymph nodes, and pleura [23]. Multiple diagnostic tests may be required in these patients for a definitive diagnosis. Concomitant ART and chemoprophylaxis do not rule out PCP and may actually contribute to more atypical presentations, highlighting the need for increased awareness of these unusual presentations when patients with HIV are receiving these treatment regimens.

## LIST OF ABBREVIATIONS

HIV: = Human Immunodeficiency Virus
OI: = Opportunistic Infections
PCP: = *Pneumocystis jiroveci* Pneumonia
ART: = Anti-Retroviral Therapy
CD4: = CD4+ T Helper Lymphocytes
TMP/SMX: = Trimethoprim/Sulfamethoxazole
CT: = Computed Tomography (CT)
IV: = Intravenous
TBBx: = Transbronchial Biopsies
